# Interval Metastases After Neoadjuvant Chemoradiotherapy for Patients with Locally Advanced Esophageal Cancer: A Multicenter Observational Cohort Study

**DOI:** 10.1245/s10434-024-15890-w

**Published:** 2024-07-27

**Authors:** Charlène J. van der Zijden, Pieter C. van der Sluis, Bianca Mostert, Joost J. M. E. Nuyttens, J. Jan B. van Lanschot, Manon C. W. Spaander, Roelf Valkema, Peter Paul L. O. Coene, Jan Willem T. Dekker, Willem E. Fiets, Hendrik H. Hartgrink, Wouter L. Hazen, Ewout A. Kouwenhoven, Grard A. P. Nieuwenhuijzen, Camiel Rosman, Johanna W. van Sandick, Meindert N. Sosef, Edwin S. van der Zaag, Sjoerd M. Lagarde, Bas P. L. Wijnhoven

**Affiliations:** 1 Department of Surgery, Erasmus MC Cancer Institute, Erasmus University Medical Center, Rotterdam, The Netherlands; 2 Department of Medical Oncology, Erasmus MC Cancer Institute, Rotterdam, The Netherlands; 3 Department of Radiation Oncology, Erasmus MC Cancer Institute, Rotterdam, The Netherlands; 4 Department of Gastroenterology and Hepatology, Erasmus University Medical Center, Rotterdam, The Netherlands; 5 Department of Nucleair Medicine, Erasmus University Medical Center, Rotterdam, The Netherlands; 6 Department of Surgery, Maasstad Hospital, Rotterdam, The Netherlands; 7 Department of Surgery, Reinier de Graaf Group, Delft, The Netherlands; 8 Department of Medical Oncology, Medical Centre Leeuwarden, Leeuwarden, The Netherlands; 9 Department of Surgery, Leiden University Medical Center, Leiden, The Netherlands; 10 Department of Gastroenterology, Elisabeth Tweesteden Hospital, Tilburg, The Netherlands; 11 Department of Surgery, Zorggroep Twente, Almelo, The Netherlands; 12 Department of Surgery, Catharina Hospital, Eindhoven, The Netherlands; 13 Department of Surgery, Radboud University Medical Center, Nijmegen, The Netherlands; 14 Department of Surgery, The Netherlands Cancer Institute–Antoni van Leeuwenhoek Hospital, Amsterdam, The Netherlands; 15 Department of Surgery, Zuyderland Medical Center, Heerlen, The Netherlands; 16 Department of Surgery, Gelre Hospital, Apeldoorn, The Netherlands

**Keywords:** Esophageal cancer, Esophagogastric junction cancer, Chemoradiotherapy, Interval metastases, Palliative therapy

## Abstract

**Background:**

Despite trimodality treatment, 10% to 20% of patients with esophageal cancer experience interval metastases after surgery. Restaging may identify patients who should not proceed to surgery, as well as a subgroup with limited metastases for whom long-term disease-control can be obtained. This study aimed to determine the proportion of patients with interval metastases after neoadjuvant chemoradiotherapy (nCRT) and to evaluate treatment and survival.

**Methods:**

Patients who had cT2-4aN0-3M0 esophageal cancer treated with nCRT were identified from a trial database. Metastases detected up to 14 weeks after nCRT on ^18^F-FDG-PET/CT or during surgery were categorized as oligometastases (≤3 lesions located in one single organ or one extra-regional lymph node station) or as non-oligometastases. The primary outcome was the proportion of patients with metastases after nCRT. The secondary outcomes were overall survival (OS) and the site and treatment of metastases.

**Results:**

Between 2013 and 2021, 973 patients received nCRT, and 10.3% had interval metastases. Of 100 patients, 30 (30%) had oligometastases, located mostly in non-regional lymph nodes (33.3%) or bones (26.7%). The median OS of this group was 13.8 months (95% confidence interval [CI] 9.2–27.1 months). Of 30 patients, 12 (40%) with oligometastases underwent potentially curative treatment, with a median OS of 22.8 months (95% CI 10.4–NA). The patients with non-oligometastases underwent mostly systemic therapy or BSC and had a median OS of 9 months (95% CI 7.4–10.9 months).

**Conclusions:**

Interval metastases were detected in about 10% of patients after nCRT, underscoring the importance of re-staging with ^18^F-FDG-PET/CT for those who proceed to surgery. A favorable survival might be accomplished for a subgroup of patients with oligometastases.

The standard care for patients with potentially curable esophageal or junctional cancer consists of neoadjuvant chemoradiotherapy (nCRT) followed by esophagectomy.^1–3^ Despite trimodality treatment, 10% to 20% of patients experience distant metastases within 1 year after surgery.^2^ For these patients, esophagectomy probably has not been of benefit because recovery from surgery takes as long as 6 to 12 months, and surgery has a lasting negative effect on quality of life. Therefore, it is pivotal to carefully select patients who can be cured by surgery.

A Dutch nationwide study reported a median survival of only 10 months for patients with interval metastases after nCRT, all of which were detected during surgery.^4^ Studies using ^18^F-FDG-PET/CT after completion of nCRT but before surgery showed that in 8% to 10% of patients, distant metastases are detected.^5–7^ Therefore, most hospitals in the Netherlands have established a re-staging ^18^F-FDG-PET/CT after completion of nCRT as the standard care.

It is unclear how patients with interval metastases should be managed and whether long-term control can be achieved, especially for patients with oligometastases. Retrospective and prospective studies on synchronous and metachronous oligometastases in patients with esophageal cancer show that a 5-year survival of 25% to 30% can be achieved for a select group.^8–10^ Given the low burden of metastatic disease, it can be hypothesized that patients with interval oligometastases also may be candidates for more aggressive treatments, even with potentially curative intent.

This study aimed to determine the proportion of patients experiencing interval metastases after nCRT and the site of these metastases, and to evaluate treatment and survival for patients with oligometastases and non-oligometastases.

## Materials and Methods

### Study Design

This retrospective multicenter observational cohort study was initiated by the Erasmus MC Cancer Institute in collaboration with the hospitals that participated in the preSANO and SANO(1/2) studies. Ethical approval for this study was waived by the local Ethics Committee (Erasmus University Medical Centre Rotterdam; MEC-2022-0185) because the patients were not subjected to any medical procedure and written informed consent had already been provided for the prospective preSANO and SANO(1/2) studies. The study was performed in accordance with the Declaration of Helsinki (64th World Medical Association General Assembly, Fortaleza, Brazil, October 2013).

### Patients

Operable patients with a resectable esophageal of junctional tumor who underwent nCRT and met the inclusion criteria of the preSANO and SANO(1/2) studies were selected from a trial database.^11–13^

Neoadjuvant chemoradiotherapy consists of five weekly cycles of carboplatin administered intravenously at an area under the curve (AUC) of 2 mg/ml/min and paclitaxel administered intravenously at a dose of 50 mg/m^2^ on the first day of each week, with concurrent radiotherapy of 41.4 Gy given in 23 fractions of 1.8 Gy for 5 days per week, starting on the first day of each chemotherapy cycle.^3^

### Response Evaluations

The first response evaluation 4 to 6 weeks after completion of nCRT included an upper gastrointestinal endoscopy with at least four bite-on-bite biopsies of the primary tumor site and suspicious areas.^14^ If residual invasive cancer, high-grade dysplasia (confirmed by two independent pathologists), or an endoscopically non-traversable tumor was confirmed, ^18^F-FDG-PET/CT was performed before the patients underwent surgery. Esophagectomy was performed only when ^18^F-FDG-PET/CT showed no distant metastases.

The patients without residual tumor at the first response evaluation underwent a second response evaluation 10 to 12 weeks after completion of nCRT. This evaluation consisted of ^18^F-FDG-PET/CT, upper gastro-intestinal endoscopy with bite-on-bite biopsies, and endoscopic ultrasound (EUS) with fine-needle aspiration (FNA) of suspicious lymph nodes.

The patients who participated in the preSANO study underwent esophagectomy after the second response evaluation. The patients without locoregional residual disease were randomized to standard surgery or active surveillance as per SANO protocol or were offered active surveillance within the SANO-2 study.^12,13^

### Detection and Treatment of Interval Metastases

Interval metastases were defined as metastases detected during the first or second response evaluation by ^18^F-FDG-PET/CT or at the time of surgery. Metastases were categorized as oligometastases or non-oligometastases. Oligometastatic disease was defined as three lesions or fewer located in a single organ or as one extra-regional lymph node station with metastases.^15^

The patients with interval metastases were discussed in the local multidisciplinary tumor board (MTB). Treatment was categorized as therapy with curative intent or purely palliative intent as deduced from the electronic patient records. If the MTB decided on treatment followed by response evaluation, this was classified as curative-intent therapy, whereas treatment without follow-up evaluation was considered as treatment with palliative-intent.

The therapies included systemic anti-tumor therapy (i.e., chemotherapy with optional immunotherapy or targeted therapy), locoregional therapy (i.e., radiotherapy or other local treatment of metastases), or best supportive care (BSC) including endoscopic stent.

### Outcomes

The primary outcome was the proportion of patients who experienced interval metastases after nCRT and the site of these metastases. The secondary outcomes were treatment and overall survival (OS) of the patients with oligometastases and non-oligometastases.

### Statistical Analyses

Baseline characteristics were presented as frequency (%) for categorical variables and as median (minimum to maximum value) for continuous variables. For binominal outcomes, comparison was made using the chi-square test and Fisher’s exact test when appropriate. The proportion of patients with interval metastases was calculated relative to all the patients treated with nCRT. Survival and follow-up evaluation were reported in months and calculated from the date of nCRT completion to the date of death or the last day of follow-up evaluation, whichever occurred first. Survival was calculated using the Kaplan-Meier method, and the log-rank test was used to determine statistical differences between groups. Statistical significance was defined as a *p* value lower than 0.05. Statistical analysis was performed using R version 4.0.4 (www.r-project.org).

## Results

### Patients

Between July 2013 and April 2021, 973 patients were selected from the trial databases. The proportion of patients with interval metastases was 10.3% (100/973), with the majority of metastases (62.6%) detected 10 to 14 weeks after completion of nCRT. In 32% of the patients, metastases were detected during the first response evaluation, whereas in 54% of the patients, metastases were detected during the second response evaluation.

Of the 100 patients, 84 (84%) had interval metastases detected by ^18^F-FDG-PET/CT (*n* = 83) or conventional computed tomography (CT) scan (*n* = 1), and metastatic disease was histologically proven in 52 (61.9%) of these patients. In one patient, interval metastases were detected on magnetic resonance imaging (MRI) scan, performed because of hemianopsia. Metastases were identified in 15 patients (15%) during planned surgery, and subsequently no resection was performed. Patient and tumor characteristics are listed in Table 1.

**Table 1:** Patient and tumor characteristics

Characteristics	All patients	Non-oligometastatic disease	Oligometastatic disease	*p* value
	(*n* = 973)	(*n* = 70)	(*n* = 30)	
	*n* (%)	*n* (%)	*n* (%)	
Sex				
Male	784 (80.6)	60 (85.7)	22 (73.3)	0.14^a^
Age: years (IQR)	68 (61.8–73.0)	69 (64–73.3)	65 (58.5–69.0)	0.08^b^
Tumor histology				
Adenocarcinoma	780 (80.2)	63 (90)	22 (73.3)	
Squamous cell carcinoma	184 (18.9)	7 (10)	8 (26.7)	
Other	9 (0.9)	–	–	**0.03**^a^
Tumor location				
Proximal/middle esophagus	118 (12.1)	7 (10)	5 (16.7)	
Distal esophagus/EGJ	855 (87.9)	63 (90)	25 (83.3)	0.35^a^
Tumor differentiation grade				
Well-differentiated (G1)	90 (9.2)	10 (14.3)	4 (13.3)	
Moderately differentiated (G2)	383 (39.4)	22 (31.4)	13 (43.3)	
Poorly differentiated (G3)	275 (28.3)	23 (32.9)	6 (20)	
Differentiation grade cannot be assessed (Gx)	188 (19.3)	–	–	
Missing	37 (3.8)	15 (21.4)	7 (23.3)	0.55^a^
Clinical T category				
cTx	159 (16.3)	2 (2.9)	–	
cT1	5 (0.5)	–	–	
cT2	190 (19.5)	10 (14.3)	3 (10)	
cT3	601 (61.8)	56 (80)	27 (90)	
cT4	15 (1.5)	2 (2.9)	–	0.52^a^
Clinical N category				
cNx	85 (8.7)	–	1 (3.3)	
cN0	352 (36.2)	14 (20)	10 (33.3)	
cN1	340 (34.9)	30 (42.9)	5 (16.7)	
cN2	176 (18.1)	21 (30)	12 (40)	
cN3	17 (1.7)	5 (7.1)	2 (6.7)	0.07^a^

Bold indicates *p*-value < 0.05

*IQR* interquartile range, *EGJ* esophagogastric junction

^a^Chi-square *p* value

^b^Kruskal-Wallis *p* value

### Site of Metastases

**Fig. 1:**
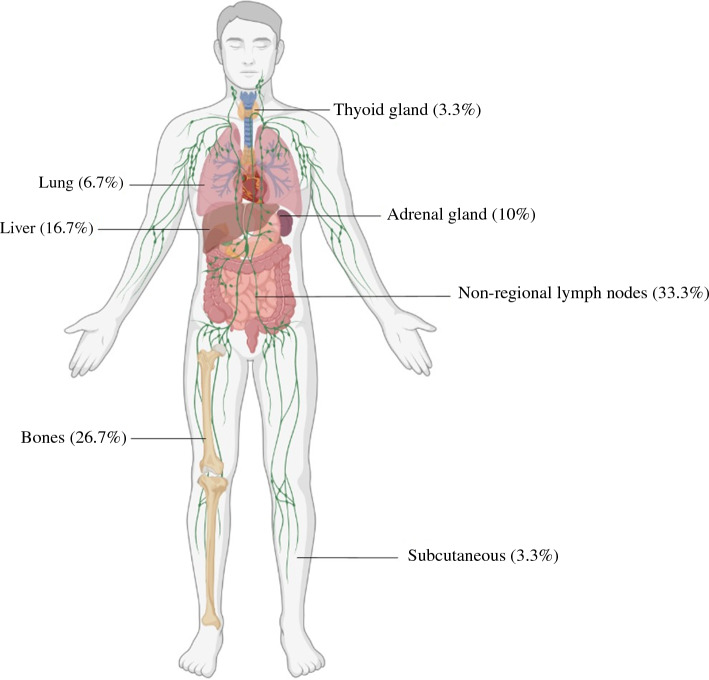
Location of oligometastases detected after neoadjuvant chemoradiotherapy (*n* = 30)

Of the 100 patients, 30 (30%) had oligometastastic disease and 70 (70%) had non-oligometastatic disease. Oligometastases were located in non-regional lymph nodes in 10 (33.3%) of 30 patients. Other locations are shown in Fig. 1. More than half of the patients (60%) with metastases detected during surgery had peritoneal metastases or organ metastases (40%).

### Treatment

**Fig. 2:**
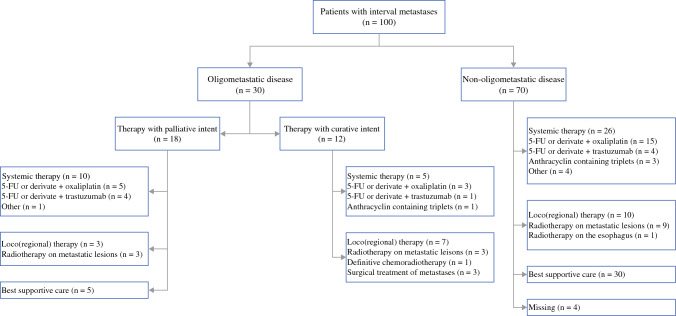
Overview of palliative and curative-intent therapy for non-oligometastatic and oligometastatic disease. 5-FU, 5-fluorouracil

More than half of the patients with oligometastatic disease (60%) underwent palliative therapy. For 10 patients, systemic therapy was performed. Only three patients were treated with palliative radiotherapy for metastatic lesions (10%), and five patients (16.7%) were treated with BSC alone. Treatment details are shown in Fig. 2.

Of all the patients with non-oligometastatic disease, 26 (37.1%) were treated with systemic therapy. Locoregional) therapy was administered to 10 patients (14.3%). No systemic or locoregional therapy was administered to 30 patients (42.9%), only BSC. Eight of these patients (26.7%) received an endoscopic stent to palliate dysphagia. For four patients (5.7%), details about palliative treatment were missing.

### Potentially Curative Therapy

Treatment with potentially curative intent was given to 12 (40%) of 30 patients with oligometastatic disease. Five of these patients underwent systemic therapy as part of induction therapy, with surgery reserved as a possibility. One patient received definitive chemoradiotherapy on metastatic lymph nodes followed by esophagectomy and cervical lymph node dissection. Three patients underwent stereotactic radiotherapy for metastatic lesions, and another three patients received surgical treatment. Two the patients who received surgery underwent resection of a metastatic lesion (located in the thyroid and adrenal glands) without esophagectomy because a clinically complete response in the esophagus was observed, and one patient underwent percutaneous radiofrequency ablation of a liver metastasis.

### Survival

**Fig. 3:**
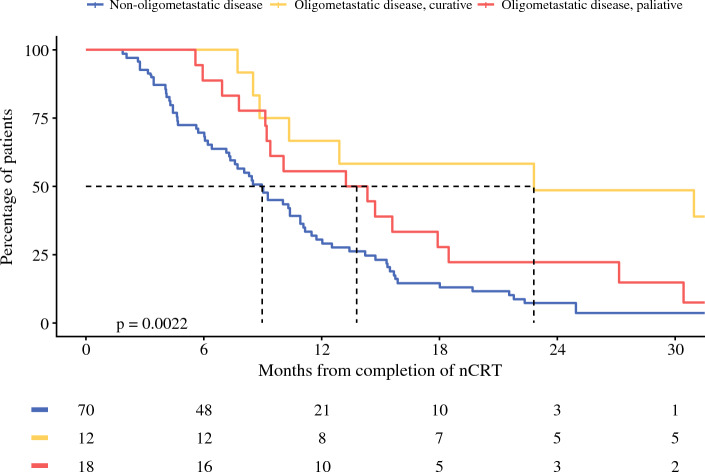
Survival of patients with non-oligometastatic disease and oligometastatic disease treated with either palliative therapy or potentially curative therapy. nCRT, neoadjuvant chemoradiotherapy

The median follow-up period for all the patients was 10 months (interquartile range [IQR], 6.0–15.7 months). The median OS of the patients with interval metastases was 10.1 months (95% CI 8.6–11.5 months). The patients with oligometastases had a median OS of 13.8 months (95% CI 9.2–27.1 months) compared with 9.0 months (95% CI 7.4–10.9 months) for the patients with non-oligometastases (*p* < 0.001). For the patients with oligometastases who underwent treatment with curative intent, the median OS was 22.8 months (95% CI 10.4–NA) compared with a median OS of 13.2 months (95% CI 4.4–22.1 months) for those treated with palliative intent (*p* = 0.13). A significant difference in OS was observed between the patients with non-oligometastases and those who had oligometastases treated with curative or palliative intent (*p* = 0.0022) (Fig. 3).

## Discussion

This multicenter observational cohort study showed that interval metastases were detected in 10.3% of patients within 14 weeks after completion of nCRT. This underscores the importance of re-staging with ^18^F-FDG-PET/CT before esophagectomy after nCRT because these patients are spared non-beneficial surgery with its inherent risks and complications as well as its lasting negative effect on quality of life.

The median OS for the patients with interval metastases in the current study (10.1 months) is comparable with the OS for patients with synchronous metastatic disease as reported previously.^16–18^ This suggests that metastases probably were already present at the time of diagnosis, but below the detection limit of ^18^F-FDG-PET/CT. Because the patients in our study underwent ^18^F-FDG-PET/CT also during initial clinical staging, the occurrence of interval metastases shortly after nCRT probably was not due to failure of clinical staging, but rather due to growth of micro-metastases. Furthermore, the proportion of detected interval metastases in our study cohort was consistent with the current literature (8%), which supports this hypothesis.^7^

The patients with non-oligometastases had a worse median OS (9 months) than the patients with oligometastases (13.8 months). This can probably be explained by the more aggressive tumor biology of patients with non-oligometastatic disease, as well as the higher metastatic burden, which results in limited treatment response and potential immunosuppression, with greater treatment challenges in the end. Additionally, the patients with squamous cell histology presented more frequently with oligometastatic than non-oligometastatic disease.^19^

On the other hand, early detection of oligometastases still may provide the option of potential curative treatment for some patients (40% in the current study). The limited number of metastatic lesions possibly creates a window of opportunity for intervention, in which induction therapy can be administered, and subsequent assessment of treatment response could aid in identifying individuals who exhibit positive responses and are suitable candidates for surgical intervention. Nevertheless, the study's small sample precluded the discernment of factors linked to the probability of qualifying for curative therapy and subsequently yielding improved survival rates.

The results of this study also point toward the need for more effective neoadjuvant treatments. Because locoregional control was achieved by chemoradiation, intensifying systemic chemotherapy may be an option. Extension of neoadjuvant therapy by adding, for example, FLOT chemotherapy or immunotherapy could be considered for treatment of micro-metastases. Previous studies combining chemotherapy with chemoradiotherapy showed improved response to chemo(radio)therapy alone.^20–22^ Ongoing trials are investigating the feasibility and safety of combined systemic therapy and locoregional therapy in oligometastatic esophageal cancer to allow for better treatment options.^23^

A strength of this study was the completeness and validity of the collected data as the patients participated in prospective studies. This made it possible to obtain detailed information on palliative treatment, including chemotherapy regimens and organ of radiation therapy. This also allowed categorization of patients between oligometastatic and non-oligometastatic disease because the number of metastatic lesions was registered.

The limitations of this study also should be mentioned. First, the different regimens of chemotherapy limit the generalizability of the results.

Second, the reason for a particular palliative treatment was not clearly defined for the majority of the patients despite access to all electronic patient records. As a result, it could be possible that certain patients themselves had decided not to opt for more intensive therapy (e.g., systemic therapy), although this was advised by the MTB. Therefore, the group of patients who underwent BSC alone and not any palliative therapy may have been overestimated with real-world data, which could have influenced the OS. Due to these lacking data, it was not possible to use this variable as a correcting factor.

Third, the short median follow-up period might have affected the survival analysis because the patients with recently diagnosed interval metastases were censored, potentially leading to less accurate survival data or an overestimation of the OS.

Fourth, this study cohort started from 2013 when only palliative chemotherapy and radiotherapy were available, before more recent target-specific tumor drivers or immune checkpoint inhibitors became available. Recent studies such as the Checkmate-648 and -649 and KEYNOTE-590 studies have shown improved survival when immunotherapy was added to chemotherapy, but this therapy was not yet available at time of this study, which was an important limitation.^24^

Finally, selection bias was present because the MTB decided whether patients qualified for potentially curative therapy. Patients’ physical condition also could have worsened at the time of interval metastases, resulting in bias because limited treatment options were available for these patients. Because no established guidelines exist to direct the therapy for oligometastases, there is room for diversity in decisions made by the MTB in this regard.

Despite these limitations, this multicenter observational cohort study provides insights into the prevalence of interval metastases after nCRT, underscoring the need of restaging before esophagectomy. Notwithstanding the dismal prognosis, a sub-selection of patients with oligometastatic disease qualify for potentially curative therapy, resulting in a favorable survival.
